# Bioinformatic Analysis of Functional Proteins Involved in Obesity Associated with Diabetes

**Published:** 2008-03

**Authors:** Allam Appa Rao, N. Manga Tayaru, Hanuman Thota, Suresh Babu Changalasetty, Lalitha Saroja Thota, Srinubabu Gedela

**Affiliations:** 1 *International Center for Bioinformatics, Department of Computer Science and Systems Engineering, Andhra University, India;*; 2 *Department of Computer Sciences and Engineering, Acharya Nagarjuna University, India;*; 3 *Annamailai University, India*

**Keywords:** bioinformatics, resistin, obesity and type two diabetes

## Abstract

The twin epidemic of diabetes and obesity pose daunting challenges worldwide. The dramatic rise in obesity-associated diabetes resulted in an alarming increase in the incidence and prevalence of obesity an important complication of diabetes. Differences among individuals in their susceptibility to both these conditions probably reflect their genetic constitutions. The dramatic improvements in genomic and bioinformatic resources are accelerating the pace of gene discovery. It is tempting to speculate the key susceptible genes/proteins that bridges diabetes mellitus and obesity. In this regard, we evaluated the role of several genes/proteins that are believed to be involved in the evolution of obesity associated diabetes by employing multiple sequence alignment using ClustalW tool and constructed a phylogram tree using functional protein sequences extracted from NCBI. Phylogram was constructed using Neighbor-Joining Algorithm a bioinformatic tool. Our bioinformatic analysis reports resistin gene as ominous link with obesity associated diabetes. This bioinformatic study will be useful for future studies towards therapeutic inventions of obesity associated type 2 diabetes.

## INTRODUCTION

Diabetes Mellitus continues to be a devastating and daunting health scourge spreading across geographical and genetic boundaries. The growing incidence of type 2 diabetes with increasing obesity reflects that obesity is an emerging risk factor for the progression of insulin resistance and subsequently to overt type 2 diabetes. Both in normoglycemic and hyperglycemic states, obese people exhibit a higher degree of hyper insulinemia that correlates with the degree of insulin resistance, in order to maintain normal glucose tolerance (2). Following attainment of certain point, the progressive deterioration of the metabolic milieu leads to eventual failure of hyperinsulinemia to compensate fully for the insulin resistance and thereby produces impaired glucose tolerance that progress to overt diabetes (5, 6). It has been presumed from genetic studies that there could be subset of genes whose expression changes with obesity and those genes whose expression further changes in the progression to type 2diabetes. However, the molecular basis that links obesity and diabetes is still largely unknown.

Despite multiple efforts are being made to dampen their impact on the quality of life of affected patients, there remains a lot of complexity exists in the pathogenesis of obesity mediated type 2 diabetes. By virtue of endocrinal role of adipose tissue, it is known to produce a vast array of adipocyte derived factors such as tumor necrosis factor alpha, interleukin-6, leptin, adiponectin and resistin. Since many of these adipokines profoundly influence insulin sensitivity and glucose metabolism, they form a fundamental bridge between increased adiposity and impaired insulin sensitivity (7). Although adipocytes are critical in obesity, their role in diabetes has been recognized.

Recently Gerken T *et al* (8) performed bioinformatic analysis and reported that the variants in the fat mass and obesity associated gene are associated with increased body mass index in humans. Barcelo-Batllori S *et al* (1) utilizes the DIGE and Bioinformatic analysis for identification of potential drug targets of tungstate, DIGE analysis identified 20 proteins as tungstate obesity-direct targets, involved in: Krebs cycle, glycolysis, lipolysis and fatty acid oxidation, electron transport and redox. Protein oxidation was decreased by tungstate treatment, which confirmed a role in redox processes; however palmitate oxidation, as a measure of fatty acid beta-oxidation, was not altered by tungstate, thus questioning its putative function on fatty acid oxidation. Bioinformatic analyses using Ingenuity pathways highlighted peroxisome proliferator activated receptor coactivator 1 alpha (PGC-1 alpha) as a potential target. Elbers CC *et al* (3) identified five overlapping chromosomal regions for obesity and diabetes. These results illustrate the importance of proteomics and bioinformatics approaches for identify new therapeutic invention of obesity is a challenging subject.

Bioinformatics has been in the focus since recent years for unraveling the structure and function of complex biological mechanisms. The analysis of primary gene products has further been considered as diagnostic and screening tool for disease recognition. Such strategies aim at investigating all gene products simultaneously in order to get a better overview about disease mechanisms and to find suitable therapeutic targets. This paper will therefore focus on potential implications of bioinformatics as a tool to identify novel metabolic patterns or markers associated with disease status. We will exemplify the potential of this method using the association between specific fats and development of obesity associated diabetes as a test case. In the present *in silico* study we have employed clustalW online bioinformatics tool for the analysis of seventeen genes, which are excepted to be play major role in obesity and diabetes, we sought to identify the common central gene/protein that connects both the metabolic disorders such as obesity and diabetes.

## METHODOLOGY

The present research aims at finding the proteins responsible for obesity associated diabetes in two phases. The first phase of the research attempts to identify the candidate proteins/genes which are involved in these disorders through thorough literature search. The data pertaining to these proteins is extracted from the databases that are available online for free access. The functional protein sequences of these proteins in FASTA are extracted from (National Center for Biotechnology Information (NCBI), (http\\www.ncbi.nih.nlm.gov).

The second phase of the research analyzes the data by employing Multiple Sequence Alignment using ClustalW online tool. These alignments produce a Phylogram tree along with the alignment scores. ClustalW adds sequences one by one to the existing alignment to build a new alignment because of its progressive nature. Progressive in this context means, it starts with using pair wise method to determine the most related sequences and then progressively adding less related sequences initial alignment.

## RESULTS & DISCUSSION

**Figure 1:**
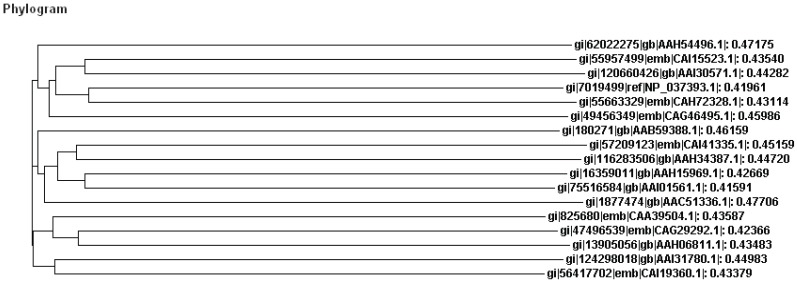
Phylogenetic tree that was constructed based on the alignment scores of all the protein sequences involved in of obesity associated with diabetes.

From thorough literature search seventeen proteins (Table 1) were collected and constructed phylogram as shown in Figure 1. From the close identification of the figure it has came to know that resistin is an important protein of obesity-associated diabetes.

**Table 1:** Showing the genes/proteins that have been studied in the present study, which are believed to be involved in type2 diabetics and obesity

S. no	Gene name	Accession number	Length	Tissue

1	*ADIPOQ*	AAH54496	244 aa	Peripheral Nervous System, sympathetic
2	*CETP*	AAB59388	425 aa	Liver
3	*HTR2C*	CAI41335	458 aa	no
4	*IAPP*	CAA39504	89aa	no
5	*ICAM1*	AAH15969	532 aa	Kidney, renal cell adenocarcinoma
6	*IL6*	CAG29292	212 aa	no
7	*LEPR*	AAI31780	232 aa	PCR rescued clones
8	*LMNA*	CAI15523	614 aa	no
9	*MAPK8*	AAI30571	427 aa	Pooled, cerebellum, kidney, placenta, testis, lung, colon, liver, heart, thyroid, bladder, uterus, PCR rescued clones
10	*PPARG*	AAH06811	477 aa	Placenta, choriocarcinoma
11	*PPARGC1A*	NP_037393	798 aa	
12	*RETN*	AAI01561	108 aa	Brain, cerebral cortex and lung, PCR rescued clones’
13	*SELE*	CAI19360	484 aa	no
14	*SLC2A4*	AAH34387	415 aa	Colon, Kidney, Stomach, adult, whole pooled
15	*SOCS3*	CAG46495	225 aa	no
16	*UCP2*	AAC51336	309 aa	skeletal muscle
17	*RBP4*	CAH72328	201 aa	

Numerous factors in obesity such as elevated free fatty acid levels, decreased adiponectin and increased adipocytokines are majorly responsible for evolution of insulin resistance (13). Resistin is a one such novel putative adipocyte derived signaling molecule induced during adipogenesis (15). It was discovered by virtue of its altered gene expression in mouse adipocytes in response to insulin sensitizers such as thiazolidinediones (TZD’s) resistin was originally named for its resistance to insulin resistin circulates as trimer and hexamer with intertrimer disulfide bond and processing of these bonds may be crucial to resistin activation (15). It is a peptide hormone that belongs to a family of tissue specific resistin like molecules (16). Since the discovery of resistin, there remains a lot of ambiguity with regard to the functional significance of resistin. Plasma resistin levels are increased in ob/ob, db/db and diet induced obese mice (15). Concomitantly resistin m-RNA levels in obese rodents are often found be decreased (12). There is often a discrepancy between circulating protein levels of resistin and m-RNA content in adipocytes (9).

In animals, resistin has been shown to be secreted by adipocytes and to impair glucose tolerance and insulin action when infused into mice. A study has also reported increased resistin expression in human abdominal tissue. Several studies, however, have reported reduced resistin expression in human and rat obesity. Insulin, FFAs, and TNF-a have all been shown to inhibit resistin expression and all of these factors are elevated in obesity. Therefore, contrasting results obtained from both human and a rodent study made the role of resistin in obesity-induced diabetes is more and more controversial. The human resistin is a dimeric protein with 108 amino acids as compared to the murine resistin which comprises 114 amino acids. It raises blood glucose and insulin concentration and reduces hypoglycemic response to insulin infusion (18). Thus it was proposed to be an important link between obesity and insulin resistance. But in human its physiological function is still debatable. This is also produced by peripheral monocytes and its level correlate with IL-6 concentration raising the possibilities that it is probably associated with inflammation induced insulin resistance.

Recently List Eo *et al* (10) performed proteomic analysis using MALDI-MS/MS and reported that 17 proteins out of 28 proteins are involved in the energy metabolism. Smith *et al* (14) study reported that a polymorphism in the promoter region was associated with resistin mRNA levels in abdominal subcutaneous fat. Associations between resistin polymorphisms and type 2 diabetes have been reported in few studies (17). On the contrary, few other studies reported no such association between resistin polymorphisms and type 2 diabetes (11). Variation in the resistin gene is associated with obesity and insulin related phenotypes in Finnish human population. The variation in the resistin gene is not directly involved in the beta cell dysfunction but it may play crucial role in the pathobiology of obesity and insulin resistance that resulted in type 2 diabetes (4). Therefore, for the first time, this bioinformatics study reinforces the role of resistin in the pathophisiology of obesity mediated insulin resistance and type 2 diabetes.

## CONCLUSION

Any rigid assessment of disease patterns will need support from well documented and curated databases. However, there are also severe practical and theoretical constraints known if applying bioinformatics as a tool for improved understanding and diagnostics of disease patterns Though lot of controversies exist with regard to the role of resistin in metabolic disorders such as obesity and diabetes mellitus, it’s role is not completely excluded. Our Bioinormatics analysis once again heightens the possible role of Resistin gene that connects obesity and diabetes mellitus. In future studies like this may pave way for new therapeutic inventions of obesity associated diabetes.
